# Bio-Based Photoreversible Networks Containing Coumarin Groups for Future Medical Applications

**DOI:** 10.3390/polym15081885

**Published:** 2023-04-14

**Authors:** Iskenderbek Elchiev, Gokhan Demirci, Miroslawa El Fray

**Affiliations:** Faculty of Chemical Technology and Engineering, Department of Polymer and Biomaterials Science, West Pomeranian University of Technology, Al. Piastów 45, 71-311 Szczecin, Poland; ei48121@zut.edu.pl

**Keywords:** photocrosslinking, dimer fatty diol, coumarin, dynamic polymer networks

## Abstract

Photocurable biomaterials that can be delivered as liquids and rapidly (within seconds) cured in situ using UV light are gaining increased interest in advanced medical applications. Nowadays, fabrication of biomaterials that contain organic photosensitive compounds have become popular due to their self-crosslinking and versatile abilities of changing shape or dissolving upon external stimuli. Special attention is paid to coumarin due to its excellent photo- and thermoreactivity upon UV light irradiation. Thus, by modifying the structure of coumarin to make it reactive with a bio-based fatty acid dimer derivative, we specifically designed a dynamic network that is sensitive to UV light and able to both crosslink and re-crosslink upon variable wave lengths. A simple condensation reaction was applied to obtain future biomaterial suitable for injection and photocrosslinking in situ upon UV light exposure and decrosslinking at the same external stimuli but at different wave lengths. Thus, we performed the modification of 7-hydroxycoumarin and condensation with fatty acid dimer derivatives towards a photoreversible bio-based network for future medical applications.

## 1. Introduction

Dynamic polymer networks, also known as smart materials, have received significant attention in recent years due to their potential applications in various fields, such as medicine, electronics, and environmental science [1,2,3,4]. These networks can change their properties in response to external stimuli, such as light, temperature, pH, and others, and are particularly interesting for biomedical applications, including drug delivery or intraocular lenses [5,6,7]. Photosensitive hydrogels are a type of dynamic polymer networks that can change their properties when exposed to light [4] and have been the focus of intense research in recent years due to their potential in controlled drug delivery and tissue engineering applications [8,9].

Coumarin is a molecule widely used in the field of photosensitive hydrogels [8,9,10,11]. This photosensitive molecule can be incorporated into the polymer backbone or attached as pendant groups, creating materials that can change their properties when exposed to the light. The inclusion of coumarin groups in the polymer network allows for external control of network properties, such as mechanical strength, swelling, degradation rate, and others [12]. Moreover, photosensitive coumarin derivatives have the advantage of being able to covalently link and unlink through exposure to different UV irradiations in a repeatedly reversible manner, thus offering the possibility of various biomedical applications, such as wound healing, drug delivery, tissue engineering, and others [13].

The introduction of coumarin groups into the polymer network can be achieved by various methods, such as chemical conjugation, physical adsorption, or incorporation into the polymer backbone [14]. The choice of method depends on the desired properties of the final material and the application for which it is intended. The use of coumarin-containing polymers, especially hydrogels, have been the main focus in recent decades [15,16]. Photosensitive hydrogels have a number of advantages over traditional hydrogels [13], including the ability to control the mechanical properties and rate of degradation of a material when exposed to light [17,18]. Other material properties, such as shape or optical properties, can also be reversibly adjusted using light energy [19].

In addition, the use of bio-based monomers for the production of new polymeric materials to replace unsustainable resources is becoming a mainstream trend of the times, and also includes polymers for medical applications. Vegetable oils are typically among the promising renewable resources, with high versatility in modifications and reactions. Dimerization of fatty acids, mainly C18 vegetable unsaturated fatty acids, like oleic or linoleic acid, is triggering increasing attention in sustainable polymer chemistry. Subsequent hydrogenation or reduction of C18 fatty acids yields difunctional monomers: fatty dimer diols or fatty dimer acids characterized by low glass transition temperature, amorphous structure, and high conformational flexibility of aliphatic chains, thus making them suitable for synthesis of various elastomeric polymers, including polyesters [20], polyurethanes [21], and flexible polymers networks [22].

The aim of this study is to investigate the potential of using bio-based fatty acid derived dimer diol and 7-hydroxycoumarin for the preparation of photoreversible networks containing coumarin groups for future medical applications, given that both raw materials are non-toxic and bioactive [23]. Moreover, the use of far UVC light in the 200–222 nm range, which is effective in inactivating pathogens while causing minimal damage to human skin [24,25], would be an added benefit of using such systems. The study includes the synthesis procedure, characterization of the obtained material by the nuclear magnetic resonance (NMR) spectroscopy and Fourier transform infrared (FTIR) spectroscopy, as well as the dynamic network formation and its sensitivity to UV light. The results obtained in this study provide information on the potential of highly efficient synthesis of photoreversible networks containing coumarin groups.

## 2. Materials and Methods

### 2.1. Materials

5-Hydroxyisophtalic acid, 7-hydroxycoumarin, ethyl bromoacetate, thionyl chloride, and potassium carbonate were purchased from Sigma Aldrich (Poznan, Poland). Fatty acid dimer diol (PRIPOL^TM^ 2033) was kindly provided by Cargill Bioindustrial (Gouda, The Netherlands). Anhydrous pyridine, anhydrous tetrahydrofuran, ethanol, acetone, hydrochloric acid, and dimethyl sulfoxide were purchased from CHEMPUR (Piekary Śląskie, Poland). Toluene was purchased from the P.P.H. Stanlab (Lublin, Poland). 1,4-Dioxane and n-hexane were provided by WarChem (Warsaw, Poland). Sodium hydroxide was purchased from PEQUINSA (Navarra, Spain). Finally, diphenyl ether was provided by VladaChem GmbH (Malsch, Germany).

### 2.2. Synthesis of 7-Carboxymethoxycoumarin

**Scheme 1:**
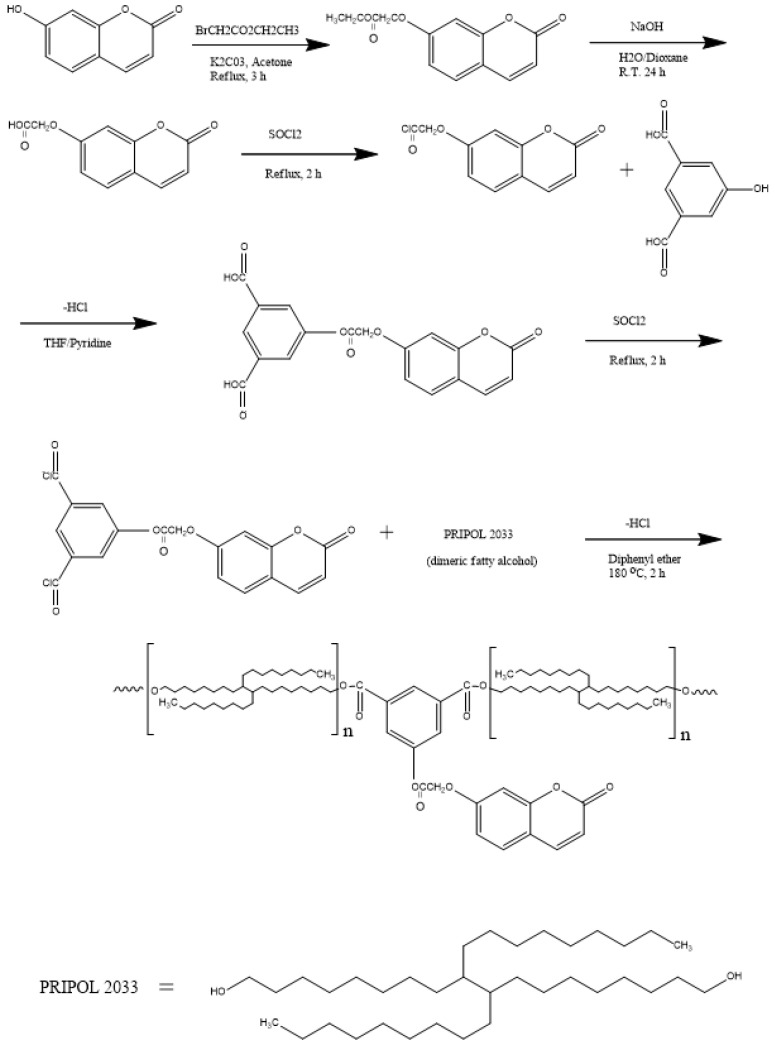
Reaction scheme of a photosensitive fatty acid dimer ester containing a pendant coumarin group.

Synthesis of the photosensitive coumarin-containing fatty acid dimer diol is presented in Scheme 1. The reaction procedure was as follows: for functionalization of the solution, 7-hydroxycoumarin (5.0 g), ethyl bromoacetate (4.18 mL), potassium carbonate (6.25 g), and acetone (150 mL) were weighed and mixed in the reflux system. Upon reaching 60 °C to exceed boiling point of acetone, the solution was refluxed for 3 h. The resulting salt was filtered, and then was recrystallized from ethanol. The obtained product was hydrolyzed in a mixture of water (200 mL), 1,4-dioxane (140 mL), and sodium hydroxide (8.10 g) at room temperature for 18 h. Acidity of the obtained product was stabilized with hydrochloric acid and in 1 h the solution became cloudy. The precipitation process was initiated by heating the mixture up to 100 °C. The cloudiness of the mixture disappeared after 20 min, turning the color to transparent yellow, after which the product had precipitated. The solid product alongside ethanol was placed into a beaker and, while maintaining a temperature of 65 °C, ethanol dissolved most of the solid material, resulting in the whole liquid mixture having a transparent vivid red color. After the recrystallization from the ethanol, the formed solid material was dissolved in an excess amount of thionyl chloride for 3 h. The obtained solution was refluxed at temp 75 °C for 3 h, after which thionyl chloride was filtered off. The residual product was recrystallized from dry toluene. 7-Chlorocarbonylmethoxycoumarin was weighed—the yield was 95%.

### 2.3. Synthesis of 7-(3,5-Dicarboxyphenyl) Carbonylmethoxycoumarin (ICM)

A mixture of 5-hydroxyisphtalic acid (1.83 g) and anhydrous pyridine (15 mL) was poured into a 3-necked round-bottomed flask. Next, the 7-chlorocarbonylmethoxy-coumarin (2.39 g) and anhydrous tetrahydrofuran (20 mL) were introduced dropwise at room temperature and stirred at a rate of 100 rpm for 60 min. The mixture was heated up to 60 °C and stirred for 2 h. The mixture was poured into the beaker with cold water and left overnight. A brownish solid was formed on the surface of the mixture. The filtered solid material was collected and placed into the oven to evaporate water. The product was refluxed with thionyl chloride (20 mL) for 3 h at 80 °C. A solution was left overnight to evaporate the thionyl chloride. The resulting crystalline product was dissolved in diphenyl ether at 35 °C.

### 2.4. Condensation Reaction

A mixture of 1 mmol of the obtained ICM dichloride (0.4 g), diphenyl ether, and PRIPOL 2033 (3.6 g) was introduced into a 3-necked round-bottomed flask under dry nitrogen flow. The temperature was raised to 170 °C in 30 min and the mixture was heated at 170–180 °C for 2 h. The solution was poured into cold n-hexane. The polymer precipitated from the mixture was filtered off and left in the oven to evaporate residual liquids.

### 2.5. Photoreversible Reaction

**Figure 1:**
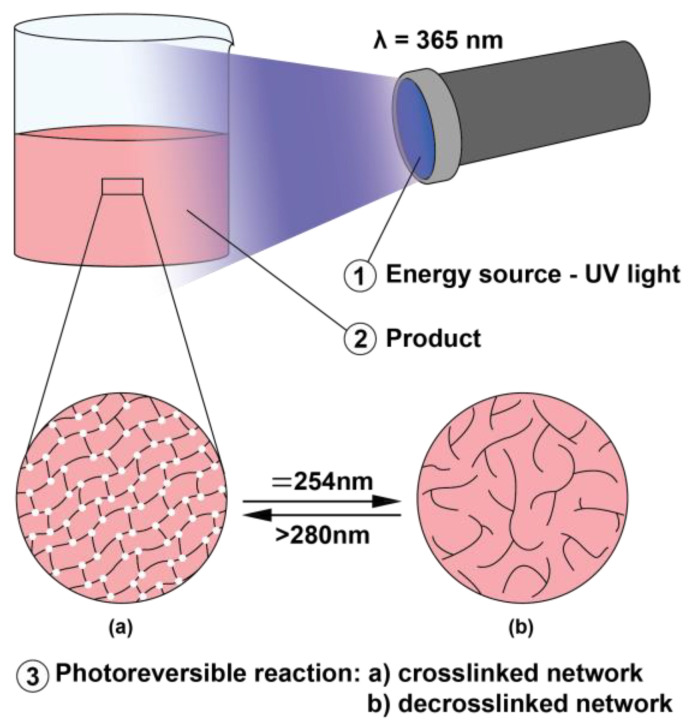
Schematic illustration of the photoreversible process of crosslinking and decrosslinking of a network.

Investigation of the photoreversible process is depicted in Figure 1 and was performed as follows: the final polymer containing a coumarin pendant group was irradiated with a DYMAX Bluewave LED Prime UVA (Torrington, WY, USA) light source, with a narrow spectral range and maximum intensity at a wavelength λmax of 385 nm for crosslinking reaction. The intensity of the radiation was adjusted to 20 mW/cm^2^ with the help of a radiometer, AktiPrint, Technigraf GmbH (Grävenwiesbach, Germany). Photocrosslinking was carried out in air atmosphere and the exposure time was 120 s. Then, the crosslinked network was introduced to the light, which was set manually with a low-pressure lamp by Heraeus and a low-pressure mercury lamp (TNN 15/32, Power: 15 Watt) with a spectrum wavelength of 254 nm for reverse photopolymerization or de-crosslinking.

**Figure 2:**
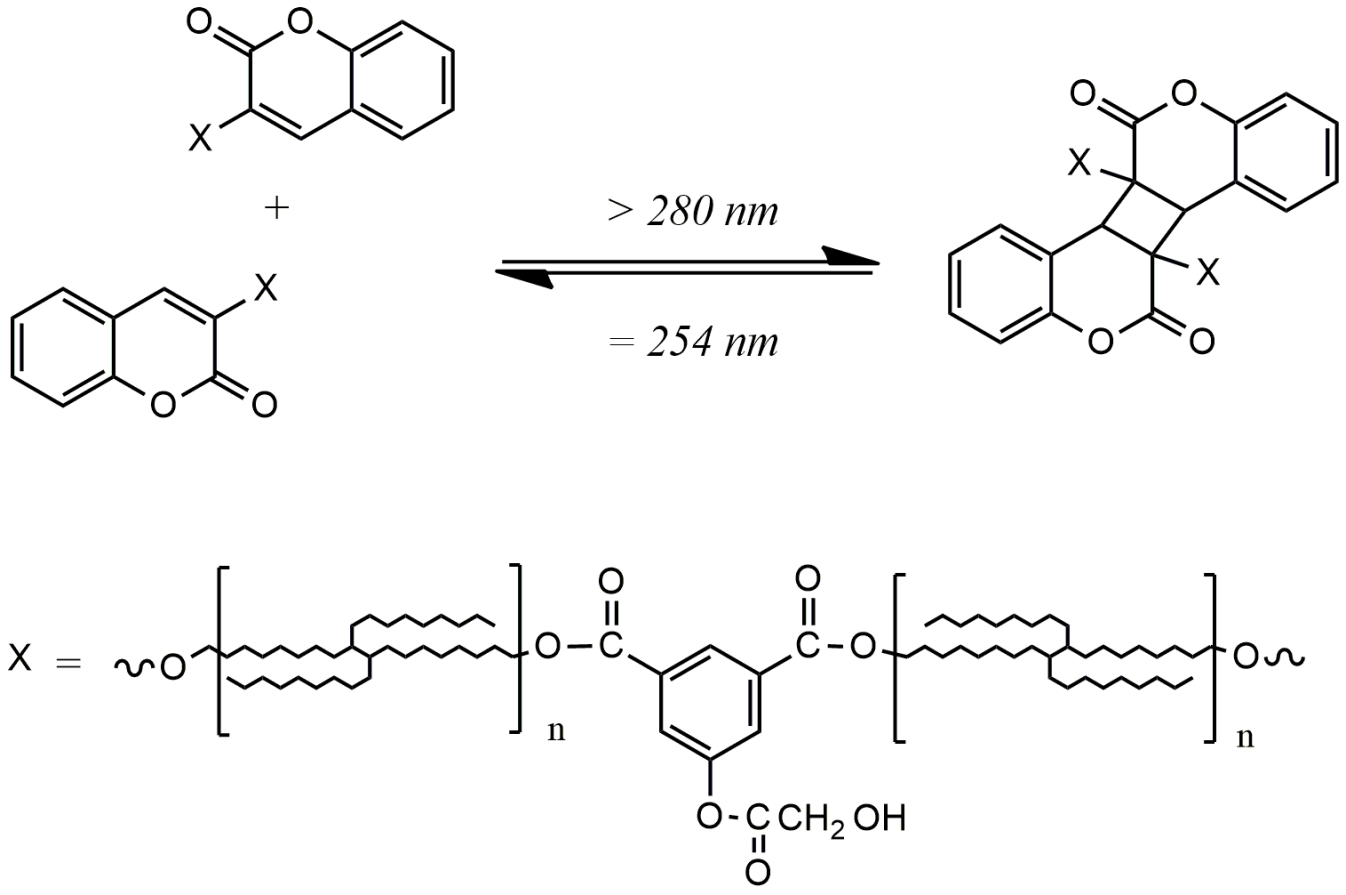
Photochemical [2 + 2] cycloaddition of coumarin-containing fatty acid dimer diol.

Coumarin moieties underwent a reversible [2πs + 2πs] cycloaddition reaction upon irradiation with specific wavelengths in the UV region, which gave reversible properties to polymers. The [2 + 2] cycloaddition reaction of the obtained polymer containing the coumarin pendant group is presented in Figure 2.

### 2.6. Characterization Methods

Fourier transform infrared spectroscopy (FTIR) was performed by using the BRUKER ALPHA Platinum apparatus (Bremen, Germany) at room temperature in the range of 4000–600 cm^−1^, at a resolution of 2 cm^−1^, and using 32 scans. Liquid (viscous) polymers were analyzed in transmission mode, after pouring samples between NaCl plates. Spectra of films after photocrosslinking were obtained using reflection mode and the ATR snap-in with the diamond crystal. Spectra were analyzed using EZ OMNIC v7.3 software.

Nuclear magnetic resonance (NMR) spectra of all obtained polymers were recorded using Bruker DPX HD-400 MHz. The instrument was equipped with a 5 mm Z-gradient broadband decoupling inverse probe. All experiments were conducted at 25 °C. Samples for NMR were prepared by dissolving approx. 20 mg of polymers in 0.7 mL of CDCl_3_-*d* and DMSO-*d*_6_.

Differential scanning calorimetry analysis (DSC) was performed using a Q2500 DSC (TA Instruments, New Castle, Delaware, USA) calorimeter to examine phase change behavior of crosslinking formulation. Samples were weighed (~5 mg) into aluminum pans and hermetically sealed before the analysis. Samples were cooled down to −90 °C, held isothermally for 10 min, and then heated up to 100 °C. The cooling/heating rate was 5 °C/min. Modulation amplitude of temperature was 0.80 °C for 60 s. The data were analyzed using TRIOS software.

Dynamic viscosity of the crosslinked formulation and after de-crosslinking was assessed using a DV3TRV rotary cone-plate rheometer, Brookfield AMETEK (Middleboro, MA, USA). The parameters for the measurement were as follows: measuring head in the cone-plate system with a diameter of ϕ = 40 mm, distance between cone and plate h = 1 mm, deformation of 30%, constant shear rate $\overset{․}{\gamma }$ (1/s) 0.200, at 25 °C.

## 3. Results and Discussion

### 3.1. FTIR Spectroscopy

**Figure 3:**
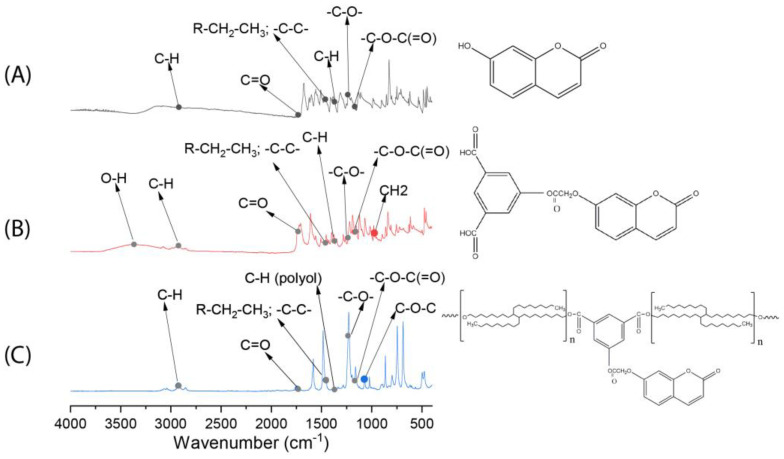
The key FTIR spectra of 7−hydroxy−coumarin (**A**), ICM (**B**), and the final fatty acid dimer ester containing the pendant coumarin group (**C**).

The FTIR spectroscopy was used to confirm chemical structure of the final product after reacting the fatty acid dimer diol with the coumarin derivative. FTIR spectra of the key molecules are presented in Figure 3.

The FTIR spectrum (Figure 3A) represents key band characteristics for 7-hydroxycoumarin used as a starting compound for ICM preparation. A broad band at 3157 cm^−1^ corresponds to -OH stretching, while the intense band at 1680 cm^−1^ can be ascribed to -C=C [26]. The bands at 1598–1614 cm^−1^ correspond to isolated and conjugated C=C bonds [27]. Characteristic C-O stretching was found at 1139 cm^−1^, while the distinct band at 826 cm^−1^ can be ascribed to out-of-plane bending vibration of C-H in the benzene ring [28,29]. The transformation of 7-hydroxycoumarin to 7-carbonyl-methoxycoumarin (ICM) has resulted in a distinct band at 1710 cm^−1^, corresponding to C=O stretching of a new ester bond and the presence of aliphatic stretching of -CH_2_- at 2924 cm^−1^ [30]. Two pendant carboxylic groups of ICM can be identified through the appearance of -OH stretching at 3385 cm^−1^ and -OH bending at 980 cm^−1^, along with C=O stretching at 1607 cm^−1^, as indicated in Figure 3B [31,32]. Finally, after introducing the PRIPOL 2033, the formation of the ester bond at 1738 cm^−1^ and aliphatic stretching of -CH_2_- at 2924 cm^−1^ from the long aliphatic chain of fatty acid can be seen, as shown in Figure 3C [30]. Moreover, distinct bands at 1581 cm^−1^ and 1480 cm^−1^ correspond to skeleton deformation vibration of the aromatic ring, while C-O stretching was found at 1230 cm^−1^, thus confirming successful bonding between ICM and PRIPOL 2033 [33].

### 3.2. NMR Spectroscopy

**Figure 4:**
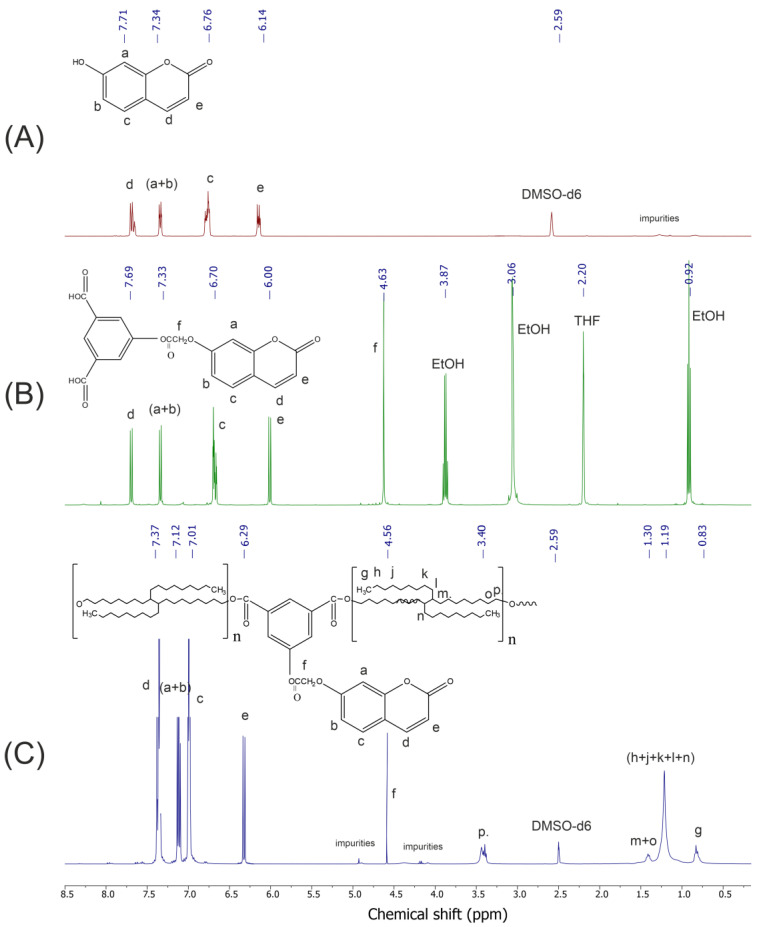
NMR spectra of 7−hydroxy−coumarin (**A**), ICM (**B**), and the final product the containing fatty acid dimer diol (**C**).

Nuclear magnetic resonance spectroscopy (NMR) technique was also used for characterization of raw molecules and resulting product. The analysis of NMR spectra also confirmed the chemical structure of synthesized compounds. All chemical shifts and their assignments are shown in Figure 4. In the ^1^H NMR spectrum, the resonances in the interval of 7.37–6.29 ppm (a, b, c, d, e) can be related to the aromatic protons present in the coumarin molecule [34]. The signal at 4.63 confirms the creation of the -OCOCH_2_O groups after the attachment of 5-hydroxyisphtalic acid to the 7-chlorocarbonylmethoxy-coumarin [35]. Furthermore, due to the introduction of PRIPOL 2033, the signals at 1.30 ppm (m, o) correspond to the tertiary carbon protons and the methylene units attached to the -CH_2_OH group. The triplet at 3.40 ppm (p.) is due to the methylene units attached to the -OH terminal group. Resonances at 1.19 ppm (h, j, k, l, n) correspond to methylene protons attached to the methyl group and the rest of methylene protons, and resonances at 0.83 ppm (g) correspond to methyl protons [36]. Number average molecular weight (M_n_) was calculated as 4450 g/mol according to NMR spectrum.

### 3.3. Differential Scanning Calorimetry (DSC)

**Figure 5:**
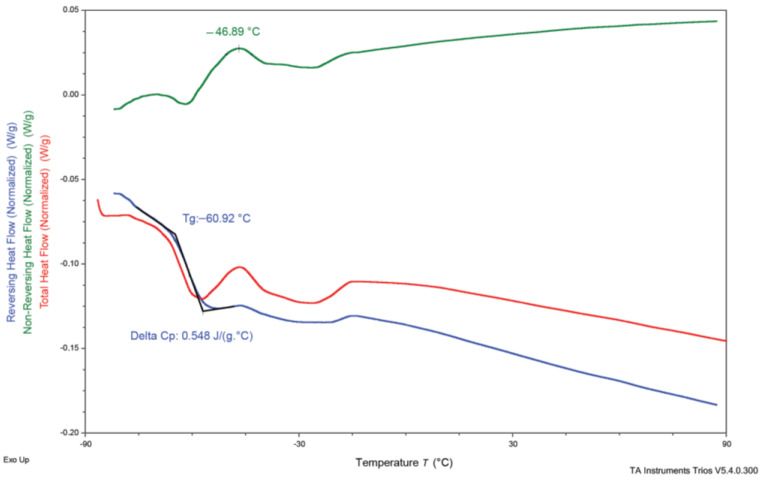
DSC thermogram of the neat formulation.

DSC was used to determine the phase transitions of the neat formulation, crosslinked, and de-crosslinked material. Due to the stress relaxation of the sample that is close to T_g_ value, modulated DSC was applied by using TRIOS software. A DSC thermogram of the neat material is presented in Figure 5, for which the glass transition temperature (T_g_) and change in heat capacity (ΔC_p_) before the crosslinking were found to be −60.9 °C and 0.548 J/g·°C, respectively.

**Figure 6:**
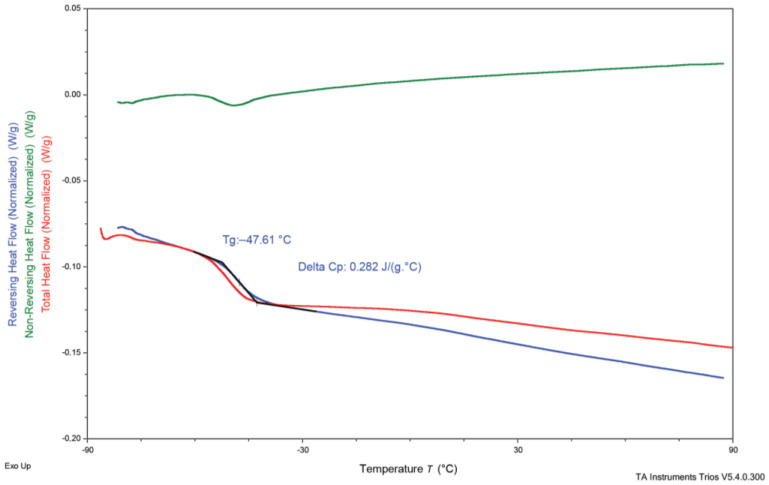
DSC thermogram of the crosslinked material.

In the case of crosslinked material (Figure 6), the glass transition temperature (T_g_) and change in heat capacity (ΔC_p_) were found to be −47.6 °C and 0.282 J/g·°C, respectively. As can be seen from the thermogram, the T_g_ shifted from −60.9 °C to −47.6 °C. The observable shift of T_g_ compared to the neat material and the relaxation stress of the crosslinked material that is visible on the non-reversing curve also confirms the successful crosslinking.

**Figure 7:**
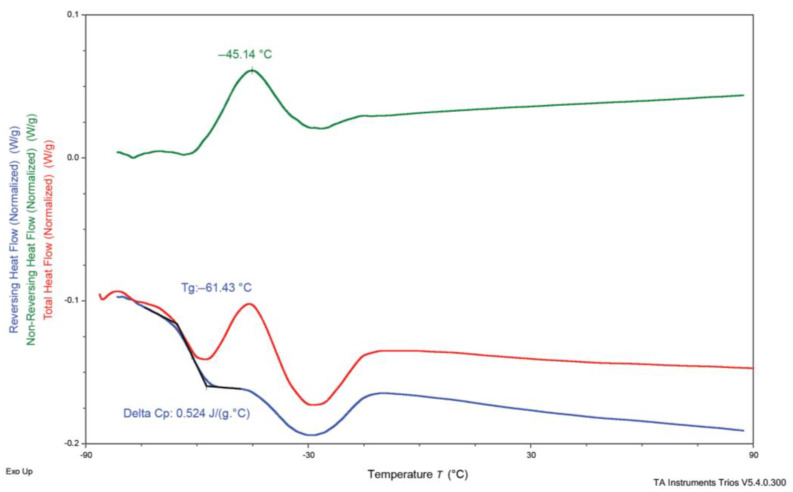
DSC thermogram of the decrosslinked material.

In the case of de-crosslinked material (Figure 7), the glass transition temperature (T_g_) and change in heat capacity (ΔC_p_) for the de-crosslinked formulation were found to be −61.4 °C and 0.524 J/g·°C, respectively. The similarity of T_g_ and ΔC_p_ values between the neat formulation and the de-crosslinked material confirm the successful reversible photo-polymerization of the polymer containing the coumarin pendant group.

### 3.4. Rheology

Dynamic viscosity of the formulation and after de-crosslinking of the polymer was performed for the investigation of the de-crosslinking of the polymer containing the coumarin pendant group. The data are collected in Table 1. It can be clearly seen that similar values of the viscosity indicate a successful de-crosslinking of the obtained polymer after irradiation at 254 nm. However, it should be mentioned that the slight increase of the viscosity could be the reason for not fully de-crosslinking the polymer.

### 3.5. Photo-Reversible Crosslinking

**Figure 8:**
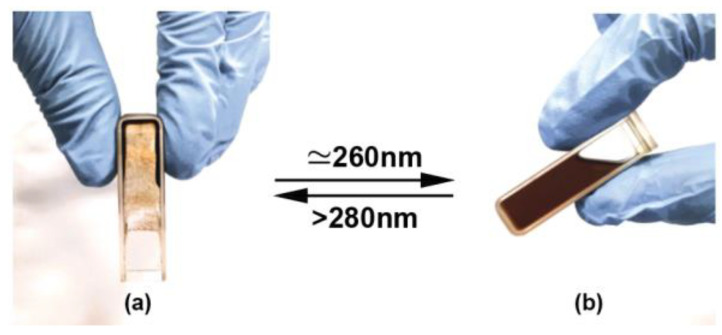
Photoreversible reaction: (**a**) crosslinked network; (**b**) de-crosslinked network.

As the photocrosslinking of the product was performed, it was possible to observe the formation of the dynamic polymer network that showed high responsiveness to the light source. At a wavelength of 385 nm, the product crosslinked as shown in Figure 8a, which is the key evidence of the reaction. Upon changing the wavelength to 254 nm, the product, on the contrary, would again come back to its original liquid state, which is also referred to as the de-crosslinked network shown in Figure 8b. In other words, due to the presence of the photosensitive coumarin group in the obtained product, it was possible to manipulate the physical state of the network by changing the wavelength of the light source. The observed phenomena describe the ability of the coumarin group to enter into a photochemical reaction, given that it is well-known that it undergoes 2 + 2 photocycloaddition [37]. Therefore, insolubility of the crosslinked network was observed due to the cyclobutane formation of coumarin dimerization upon the UV irradiation at 385 nm.

## 4. Conclusions

A photosensitive fatty acid dimer diol derivative containing coumarin was synthesized successfully and characterized by FTIR and NMR spectroscopies. The responsiveness of the product to the UV light source of different wavelengths was observed. We demonstrated—for the first time—that coumarin-containing bio-based product based on a fatty acid derivative is photosensitive in the presence of UV light at different wavelengths. Thus, the dynamic polymer network has been created, as evidenced by differential scanning calorimetry performed at modulated temperature and demonstrating successful crosslinking and decrosslinking reaction. The obtained product can be suitable for future medical applications such as coatings for medical devices, wound dressings, and surgical tools to prevent infections by inactivating pathogens while causing minimal harm to human skin, with simultaneous insolubility of the material in water as an asset. However, detailed studies related to cytotoxicity and hydrolytic and enzymatic degradation of such flexible and dynamic polymer networks are needed and under way.

## Figures and Tables

**Table 1:** Dynamic viscosity of crosslinking formulation and de-crosslinked material.

Sample	Dynamic Viscosity (mPa⋅s) at 25 °C
Before crosslinking	43,660
After de-crosslinking	45,670

## Data Availability

Not applicable.
